# Comparing Circadian Dynamics in Primary Derived Stem Cells from Different Sources of Human Adult Tissue

**DOI:** 10.1155/2017/2057168

**Published:** 2017-10-23

**Authors:** Eve H. Rogers, Sandra A. Fawcett, Vanja Pekovic-Vaughan, John A. Hunt

**Affiliations:** ^1^Institute of Ageing and Chronic Disease, University of Liverpool, 6 West Derby Street, Liverpool L7 8TX, UK; ^2^School of Science and Technology, Clifton Campus, College Drive, Nottingham NG11 8NS, UK

## Abstract

Optimising cell/tissue constructs so that they can be successfully accepted and integrated within a host body is essential in modern tissue engineering. To do this, adult stem cells are frequently utilised, but there are many aspects of their environment *in vivo* that are not completely understood. There is evidence to suggest that circadian rhythms and daily circadian temporal cues have substantial effects on stem cell activation, cell cycle, and differentiation. It was hypothesised that the circadian rhythm in human adult stem cells differs depending on the source of tissue and that different entraining signals exert differential effects depending on the anatomical source. Dexamethasone and rhythmic mechanical stretch were used to synchronise stem cells derived from the bone marrow, tooth dental pulp, and abdominal subcutaneous adipose tissue, and it was experimentally evidenced that these different stem cells differed in their circadian clock properties in response to different synchronisation mechanisms. The more primitive dental pulp-derived stem cells did not respond as well to the chemical synchronisation but showed temporal clock gene oscillations following rhythmic mechanical stretch, suggesting that incorporating temporal circadian information of different human adult stem cells will have profound implications in optimising tissue engineering approaches and stem cell therapies.

## 1. Introduction

Without adult stem cells (ASCs), mammalian tissues would not be able to maintain their natural homeostasis, as these cells play a vital part in the replenishment and repair processes, whereby old or damaged cells become replaced. Stem cells are characterized by their extraordinary abilities to self-renew through cell division over a long period of time and to be able to differentiate and give rise to organ- or tissue-specific cells in response to internal or external stimuli. It is these homeostatic and regenerative abilities that allow them to regularly repair and replace damaged or injured tissues. *In vivo*, a combination of chemical, biological, and physical cues present in the stem cell niche contribute to the direction of mesenchymal stem cell (MSC) fate, which allows them to have such a broad multilineage differentiation potential.

Recently, there has been a drive to investigate the use of Oct-4 expressing dental pulp-derived mesenchymal-like stem cells (DPSCs) in tissue engineering, as the differentiation capacity of MSCs may be restricted by their tissue of origin. These newly discovered cells offer an alternative to bone marrow-derived MSCs (BMSCs), as they have been shown to have a more potent differentiation potential than BMSCs. DPSCs have been shown to have a faster proliferation rate as well as the potential to differentiate into not only several mesenchymal cell types but also neurogenic cells, as well as being highly advantageous in terms of accessibility [1]. DPSCs are able to differentiate into neural cells as they originate from the migrating neural crest cells during embryogenesis. Dental pulp therefore consists of ectomesenchymal elements, containing neural crest-derived cells that exhibit both plasticity and multipotency [2]. The stem cells that reside within the dental pulp are extremely protected from external stimuli in their “sealed niche.” The embryonic tissues found there remain undifferentiated within the jaws until the only organogenesis event which occurs after birth.

Another, more accessible source of adult stem cells that is currently being investigated is from the adipose tissue (ADSCs); adipose tissue offers an abundant source of MSCs which can be obtained in large quantities with minimal patient discomfort. Both DPSCs and ADSCs are more favourable to BMSCs as they also result in much lower site morbidity. All three have been investigated in terms of their differentiation capacities; for example, Davies et al. [3] found that DPSCs exhibited the highest potential to produce a mineralised matrix, but ADSCs and BMSCs showed enhanced dentinogenic and mineral volume. Stanko et al. [4] found that these three cell types showed no differences in terms of cell morphology or MSC surface marker expression. However, they did find significant differences regarding the expression of several pluripotency genes; BMSCs and ADSCs produced similar protein levels of several pluripotency markers but the DPSCs showed significant differences in the amount of protein products observed, including a lower expression of Oct3/4. These differences were hypothesised to reflect the mixed embryonic stem cell origin of DPSCs.

One temporal cue recently discovered to regulate mesenchymal stem cell potential and differentiation capacity is governed by the circadian rhythm [5–7]. The mammalian circadian rhythms are orchestrated by a hierarchy of self-sustained tissue oscillators. The suprachiasmatic nucleus (SCN) in the anterior hypothalamus of the brain coordinates a number of peripheral tissue oscillators to regulate a coherent rhythm of a multitude of outputs regulating metabolism, physiology, and behaviour [8]. The molecular mechanisms that regulate the circadian clockwork are evolutionarily conserved and cell-autonomous, whereby a network of autoregulatory transcriptional-translational feedback loops drive circadian expression patterns of the core clock components [9]. The primary transcriptional-translational feedback loop (TTFL) is controlled by the basic helix-loop-helix transcription factors CLOCK and BMAL1 (i.e., ARNTL). When these two proteins heterodimerize, they are able to bind to *cis*-regulatory enhancer elements within target core clock genes as well as many clock-controlled genes (CCGs) [10, 11]. Core clock genes include *period (Per1, Per2*, and *Per3)* and *cryptochrome* (*Cry1* and *Cry2*); these two proteins also heterodimerize and repress their own transcription by negatively regulating the CLOCK:BMAL1 complex [12, 13]. The CLOCK:BMAL1 heterodimers also regulate the transcription of retinoic acid-related orphan nuclear receptors, REV-ERBs (i.e., NR1D1 and NR1D2) and RORs, which form part of the stabilising loop. These are known to bind to retinoic acid-related orphan receptor response elements (ROREs), which are present in the *Bmal1* (i.e., *ARNTL*) promoter. REV-ERBs repress transcription of *Bmal1*, whereas RORs activate the transcription [14]. Both positive and negative autoregulatory loops constitute a circadian molecular clock and take approximately 24 hours to complete.

A recent field of investigation has shown that the clock genes can directly influence ASC and progenitor cell activation and differentiation, within their tissue-specific niches. For example, disrupting the clock gene *Bmal1* leads to increased adipogenesis, and thus the attenuation of Bmal1 expression *in vitro* in preadipocytes leads to a downregulation of the Wnt signalling pathway and increased adipogenesis [15]. However, in mature adipocytes, it has been found that BMAL1 is highly expressed in differentiated cells; when BMAL1 was knocked-down by RNA interference, the mature 3T3-L1 cells were only able to accumulate minimum amounts of lipid droplets in the cells. Furthermore, adenovirus-mediated BMAL1 expression resulted in the induction of several factors involved in lipogenesis, many of which showed clear circadian rhythm in mice adipose tissue [16]. This conflicting research shows that the circadian rhythm has a clear role in cell differentiation and may have differential roles in the different stages of differentiation, but this still requires extensive further research.

The involvement of circadian clocks in the regulation of adult stem cell activation is not only niche-specific but can also act at the cell population level. Janich and colleagues showed that the circadian clock has a differential role in regulating the activation of coexisting epidermal stem cell populations. The authors found that the genes regulating stem cell niche dormancy, activation, and differentiation contained several putative BMAL1/CLOCK*-*binding sites. Deleting *Bmal1* leads to circadian arrhythmia, decreased expression of Wnt-related genes, and TGF-*β* inhibitors and causes progressive accumulation of dormant stem cells. Deleting *Per1/2*, conversely, results in progressive depletion of dormant stem cells [5]. A subsequent study showed that the stem cells responded differently to differentiation cues at certain times of the day. Interestingly, different proliferation-related or differentiation-related genes were expressed at different times of the day; for example, DNA replication and cell division related pathways were highly expressed in the dark phase, as opposed to in the light phase, when differentiation pathways were more highly expressed [6].

The endogenous mammalian circadian clock has a period of approximately 24 hours, which is reset daily by external cues, known as zeitgebers. The most potent of these is a daily light cue, which entrains the clock in the SCN in mammals through reticulo-hypothalamic signalling mechanisms. The SCN relays this information to peripheral tissue clocks via diffusible neuro-endocrine signals [17]. Another important timing signal which has the capacity to entrain daily rhythms is via systemic factors such as growth factors and hormones. Glucocorticoids, which are a class of steroid hormones that bind to the glucocorticoid receptor (GR) present in almost every vertebrate cell, have been implicated in synchronising peripheral circadian rhythms. A recently discovered entrainment mechanism for the clock that requires fundamental research is by mechanical stimulation, whereby cells in different tissues in the body are subjected to very different levels of mechanical strain. Mechanical vibrations have the capability of resetting the clock in *Drosophila melanogaster*; it has been demonstrated that rhythmic mechanical stimulation of the chordotonal organs can synchronise the *Drosophila* circadian clock. *Drosophila* with loss of function mutations in their period gene did not exhibit this ability of synchronization through vibrations, highlighting the importance of functional clock systems for mechanical entrainment [18].

Previous extensive work in MSCs has demonstrated significant effects that mechanical forces *in vitro* can exert on their proliferation and differentiation properties. For example, Tirkkonen et al. [19] used vibrational loading to cause the differentiation of human ADSCs towards bone-forming cells and inhibition of adipogenesis. The authors found that the hADSCs cultured in osteogenic conditions were sensitive to vibrational loading, and their osteogenic differentiation was enhanced with high-frequency vibration. It has also been recently published that the mechanical environment of the epithelial stem cell niche within the mammalian mammary tissue controls the amplitude of the molecular clock oscillations, which is altered upon environmental (e.g., ageing) and genetic clock disruption [7].

Interestingly, unlike adult stem cells such as BMSCs and ADSCs, which are capable of circadian synchronization by hormonal and growth factor signals such as dexamethasone [20] and serum shock [21]. Embryonic stem (ES) cells lack a ~24 h circadian rhythm and do not display the core TTFL required for circadian clock gene expression. However, upon differentiation, ES cells in culture can gain a molecular circadian rhythm, which can also be reversed when the cells are reprogrammed with the addition of *Oct3/4*, *Sox2, Klf4*, and *c-Myc* [22]. However, investigating the circadian rhythm in embryonic cells presents many challenges and the way by which we undertake our experiments may result in a disruption of endogenous oscillation(s) [23]; therefore, this lack of circadian rhythm in embryonic stem cells may not be as strict as it previously appeared.

It was hypothesised here that adult mesenchymal stem cells from different human tissue sources at different anatomical locations within the body may exhibit different circadian dynamics and respond to temporal cues differently and that the more primitive adult stem cells such as DPSCs would be less responsive to circadian synchronisation cues compared to BMSCs or ADSCs. In addition to well-established chemical synchronisation using a synthetic glucocorticoid, dexamethasone [24], mechanical stimulation was also used in a circadian paradigm to investigate whether different human adult stem cells could be entrained using mechanical cues. The applied method investigating mechanical stimulation, using a uniaxial stretch rig composed of flexible silicone substrates at 6.66% radial distension and frequency of 1 Hz, was selected as these parameters have previously found to be within physiological range. Published data has previously shown that a uniaxial strain between 5–15% with a frequency of 1 Hz is preferable for MSCs and shows positive effects on proliferation and collagen synthesis [25]. Furthermore, O'Cearbhaill et al. [26] found that radial distensions of 5% and frequencies of 1 Hz caused mechanosensitive effects including cell reorientation parallel with the direction of flow and adapted morphologies, highlighting that there is a significant cytoskeletal restructuring in these mechanically stimulated MSCs compared to static controls.

If viable, this mechanical stretch paradigm could offer a synchronisation method that excludes the need to use chemical or thermal approaches to synchronise the circadian clock, which would be hugely advantageous in tissue engineering and regenerative medicine. In this research, the circadian differences in adult stem cells derived from different human adult tissues with respect to their expression of several core clock genes, stabilising clock genes and stem cell markers in both dynamic and static conditions have been determined and compared. The results reveal differential circadian gene expression patterns in human adult stem cells derived from different tissue sources upon glucocorticoid synchronisation. It is also shown that rhythmic mechanical stimulation has the ability to entrain some human stem cells, which provides a novel clock synchronisation approach independent of chemical or temperature cues. Such a clock synchronisation protocol may prove more advantageous in future tissue engineering applications, leading to significant developments in both age-related diseases and tissue engineering and synchronising stem cell therapies.

## 2. Materials and Methods

### 2.1. Isolation of Adipose-Derived Stromal Cells

Human lipoaspirate was harvested during a lipofilling procedure after breast tumour removal. Lipoaspirate (5 g) was washed by addition of serum-free DMEM medium (Gibco, UK) and centrifuged for 3 min at 500*g*. The washed fat layer was moved to a new tube to which a digestion solution containing 10 mL of DMEM medium (Gibco, UK) and 20 *μ*L of collagenase type 2 (Sigma, UK) was added. This was placed on a roller mixer for 30 min at 37°C. 10 mL of DMEM medium containing 10% FBS and penicillin/streptomycin (Sigma, UK) was added and the tube was spun for 5 min at 1000*g*. The oil layer was removed and discarded, and undigested fat fraction was placed into a T25 falcon flask, with the addition of complete growth DMEM medium as above. The remaining supernatant was discarded and the pellet was resuspended and placed in a separate T25 flask, with the addition of complete growth DMEM medium. Both of these fractions were cultured until confluency and used at passage 2.

### 2.2. Cell Culture of BMSCs and DPSCs

Primary human BMSCs and DPSCs were obtained commercially (Lonza and BioEden Limited, resp.) and expanded in Dulbecco's Modified Eagle Medium (GlutaMAX; Gibco, UK) supplemented with 1% penicillin/streptomycin and 10% FBS and incubated at 37°C in 5% CO2. Cells were trypsinised upon reaching 70–80% confluency and used before reaching passage 7.

### 2.3. Clock Synchronisation with Dexamethasone

Cells were seeded into 6-well plates in complete growth medium as above until confluency. They were synchronised using 100 nM dexamethasone and total RNA collected every four hours over a circadian cycle at the following time points after synchronisation (h): 16, 20, 24, 28, 32, 36, 40, 44, 48, and 52. The cells were exposed to the dexamethasone for 1 h and then incubated in complete growth medium until sample collection. Negative controls received no dexamethasone but were exposed to the same media changes and conditions.

### 2.4. Mechanical Stimulation

Flexible silicone chambers were coated in fibronectin for 1 h before cells were seeded into the chambers and allowed at least 24 h to settle and attach. Once confluent, the chambers were loaded into a unique uniaxial stretch rig and stretched for 3 consecutive days in a rhythmic manner (frequency 1 Hz, 6.66% stretch, 12 h ON/12 h OFF). After day 3 of rhythmic stimulation, cells were allowed to rest and were collected at the following times over 1.5 circadian days following mechanical stimulation (h): 16, 20, 24, 28, 32, 36, 40, 44, 48, and 52.

### 2.5. Quantitative RT-qPCR

Total RNA was extracted using TRI-reagent (Sigma) as per the manufacturer's specifications. Total RNA was determined using nanodrop spectroscopy before cDNA synthesis using Superscript III Reverse Transcriptase (Invitrogen), with Oligo dT at 50°C for 40 min in a 20 *μ*l reaction. Real-time RT-PCR was carried out on cDNA samples with SYBR Green PCR Supermix (Bio-Rad) using the CFX Connect™ Real-Time PCR Detection System (Bio-Rad) under the following cycling conditions: 95°C for 3 min; 40 cycles of 95°C for 10 s; and 60°C for 30 s. Results were normalised relative to a housekeeping gene *GAPDH* expression. Primers were designed against the following genes: glyceraldehyde 3-phosphate dehydrogenase (*GAPDH*), aryl hydrocarbon receptor nuclear translocator-like (*ARNTL* or *Bmal1*), period 2 (*Per2*), period 1 (*Per1*), nuclear receptor subfamily 1 group D member 1 (*NR1D1* or *Rev-ErbAα*), and SRY (sex determining region Y)-Box 2 (*Sox2*) (primer sequences are listed in Table 1).

### 2.6. Statistical Analyses

Data were expressed as the mean ± standard error. Statistical analyses were performed, following the determination of normal distribution, using one-way analysis of variance (ANOVA), with Tukey HSD post hoc, or the independent *t*-test at a confidence level of 95% (SPSS 24 Software). In order to determine circadian gene rhythmicity and its significance, cosinor periodogram analysis was used, made available online by the Refinetti circadian biology group at Boise State University (http://www.circadian.org/softwar.html). *p* values ≤0.05 were considered statistically significant.

## 3. Results

### 3.1. Human Stem Cells Derived from Different Sources of Adult Tissue Show Differential Circadian Clock Gene Expression Profiles

**Figure 1:**
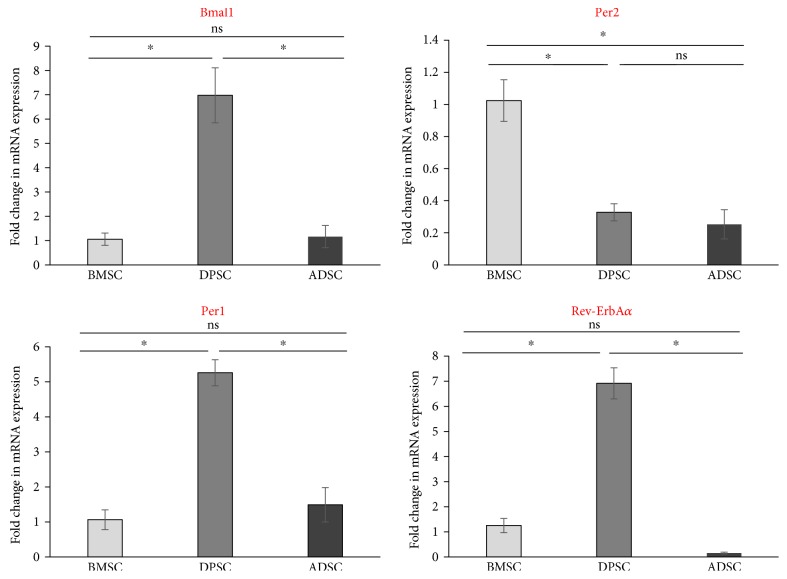
Clock gene expression in unsynchronised adult stem cells. Quantitative RT-PCR analyses comparing the relative mRNA expression levels of core clock genes in mesenchymal-like adult stem cells derived from human bone marrow, dental pulp, and adipose tissue. Data is expressed as the mean of ΔCt ± SEM normalised against the housekeeping gene *GAPDH*. Bars represent means ± SEM of 3 independent samples, ^∗^*p* < 0.05 (one-way ANOVA).

Asynchronous cultures of BMSCs, ADSCs, and DPSCs were allowed to grow to confluency in complete growth medium, before their RNA was extracted and a PCR “clock panel” of genes was used to analyse their relative mRNA expression. As expected, different human adult stem cells did exhibit significantly varying amounts of clock gene expression, with the BMSCs and DPSCs appearing to show contrasting expressions of *Bmal1*, a component of the positive arm of the molecular clock, and *Per2*, a component of the negative arm (Figure 1). Interestingly, *Bmal1* was significantly higher in DPSCs compared to BMSCs and ADSCs, whilst *Per2* was significantly higher in BMSCs compared to DPSCs and ADSCs. *Per1* and *Rev-ErbA* were also both significantly higher in DPSCs than ADSCs and BMSCs. In contrast, *Bmal1*, *Per1*, and *Rev-ErbA* were not significantly different in expression levels between BMSCs and ADSCs.

### 3.2. Glucocorticoid Stimulation Using Dexamethasone Leads to Circadian Synchronisation in BMSCs and ADSCs but Not DPSCs

**Figure 2:**
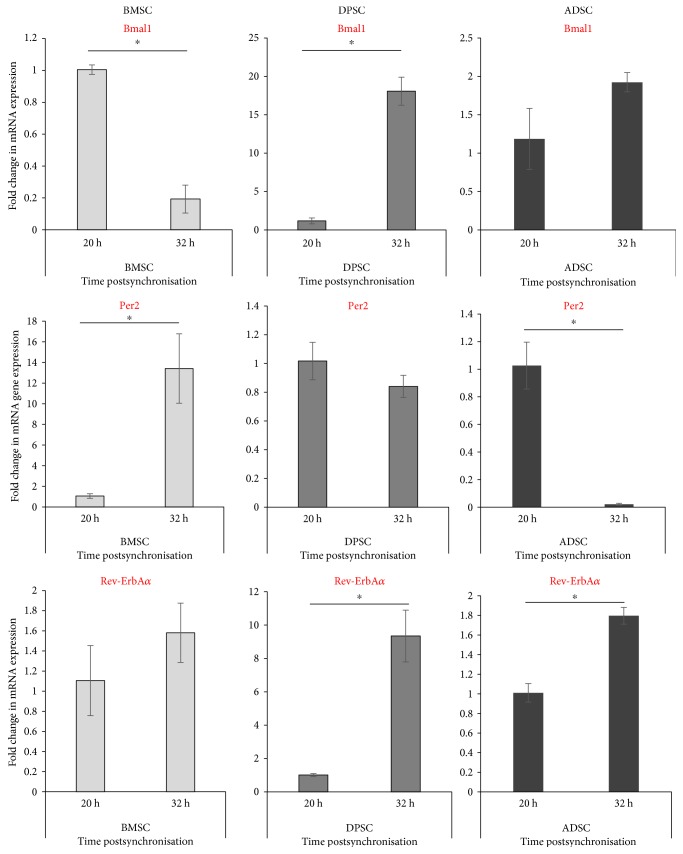
Clock gene expression in synchronised human adult stem cells at two opposite circadian phases. Quantitative RT-PCR analyses showing the expression levels of clock genes after synchronisation with dexamethasone at two opposite circadian phases 12 h apart (20 h versus 32 h). Data are expressed as the mean of ΔCt ± SEM relative to the 20 h time point and normalised against the housekeeping gene *GAPDH.* Bars represent means ± SEM of 3 independent experiments, ^∗^*p* < 0.05 (independent *t*-test).

Next, the three human adult stem cell types were grown to confluency and clock synchronised using synthetic glucocorticoid, 100 nM dexamethasone for a period of 1 h, after which, their media was replaced with complete growth medium. RNA samples were initially taken at two opposite circadian phases at either 20 h or 32 h post synchronisation to examine any initial differences at opposite phases. The antiphasic relationship of *Bmal1* and *Per2* could be clearly seen with the two genes peaking and troughing at opposite circadian times, respectively. For example, in ADSCs and DPSCs, *Bmal1* expression was much higher at 32 h than at 20 h whilst *Per2* was much higher at 20 h compared to 32 h (Figure 2). Interestingly, the opposite appeared to be observed for the BMSCs, which showed higher *Bmal1* and lower *Per2* expression at 20 h versus 32 h, respectively. The mRNA expression of a component of the stabilising loop in circadian machinery was also examined. It was found that *Rev-ErbAα* expression showed temporally different expressions at the two circadian time points in different human adult stem cell types and interestingly appeared to peak at 32 h similarly to *Per2* in BMSCs, whilst at the same circadian time point, it peaked similar to *Bmal1* in the ADSCs and DPSCs.

**Figure 3:**
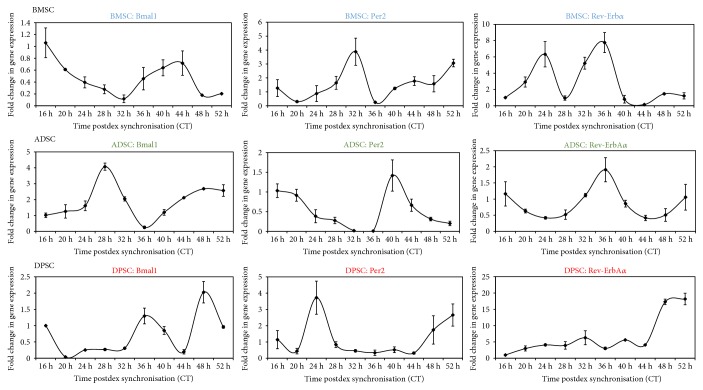
Circadian rhythm dynamics in human adult stem cells following synchronisation with dexamethasone. Quantitative RT-PCR analyses showing temporal expression profiles of clock genes collected every four hours between 16 h–52 h following synchronisation with dexamethasone. Data are expressed as the mean of ΔCt ± SEM relative to 16 h time point and normalised against the housekeeping gene *GAPDH.* Bars represent means ± SEM of 3 independent experiments.

As significant differences were observed between the two initial time points, temporal clock gene expression profiles over 1.5 circadian days were then compared in order to get a clearer understanding of the clock gene expression, omitting the first 0.5 circadian day (0–15 h) in order to exclude the transient effects of dexamethasone, as published previously [7]. Samples were collected every four hours starting with 16 h postdexamethasone synchronisation and ending at 52 h, in order to more closely investigate circadian gene dynamics between the three different human adult stem cell types (Figure 3). Both the ADSCs and BMSCs showed robust oscillations of clock gene expressions which is a characteristic antiphasic temporal pattern of synchronised cells reported previously. For example, in the BMSCs, *Bmal1* troughed whilst *Per2* peaked at 32 h postdexamethasone synchronisation. In contrast, in ADSCs, *Bmal1* peaked and *Per2* troughed around 16–20 h, showing a clear antiphasic relationship. On the other hand, the more primitive DPSCs, however, did not exhibit oscillating expressions of the circadian clock genes, and no clear temporal patterns of clock gene expression could be observed following dexamethasone synchronisation.

When analysed by cosinor periodogram (Table 2), *Bmal1* expression was shown to exhibit significant circadian rhythmicity in BMSCs (*p* = 0.007), whilst *Per2* was just short of significance (*p* = 0.076), and all three circadian clock genes (*Bmal1, Per2*, and *Rev-ErbAα*) showed circadian rhythmicity in ADSCs (*p* = 0.006, *p* = 0.021, and *p* = 0.001, resp.). However, no core clock genes were found to exhibit significant circadian rhythmicity in DPSCs, except for *Rev-ErbAα* which nearly reached significance (*p* = 0.055).

In order to confirm cell synchronisation by dexamethasone and exclude the possibility that changing the cell culture growth medium itself following dexamethasone synchronisation had any synchronising effects on cells, unsynchronised BMSCs were used as a negative control and collected temporally having received no stimulation other than the same media changes as the above timecourse experiment. As expected, no circadian rhythm of core clock genes *Bmal1* or *Per2* was observed in unsynchronised BMSCs (Supplementary Fig. 1 available online at https://doi.org/10.1155/2017/2057168).

### 3.3. DPSCs Can Be Entrained by Rhythmic Mechanical Stretch Synchronisation

**Figure 4:**
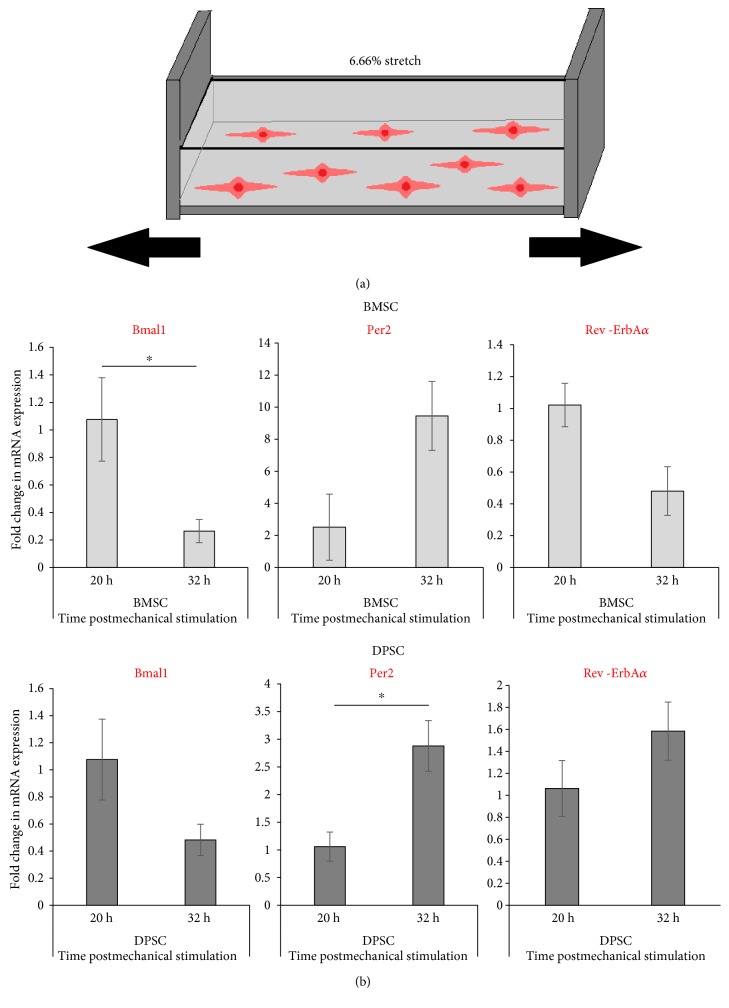
Clock gene expression in human adult stem cells following mechanical rhythmic stimulation paradigm. (a) Schematic diagram showing the setup of the mechanical stretch apparatus; cells were seeded into silicone chambers and stretched for 3 days using a unique uniaxial stretch rig with offset cams (1 Hz, 6.66% stretch, 12 h ON (stretch), 12 h OFF (rest)). (b) Quantitative RT-PCR analyses showing the expression levels of clock genes at two opposite circadian phases (20 h versus 32 h) following rhythmic mechanical stimulation protocol. Data are expressed as the mean of ΔCt ± SEM relative to the 20 h time point and normalised against the housekeeping gene *GAPDH.* Bars represent means ± SEM of 3 independent experiments, ^∗^*p* < 0.05 (independent *t*-test).

In order to find a novel method of synchronising the DPSCs, it was hypothesised that as these stem cells are encapsulated in a tight niche in the tooth but are still subjected to a substantial amount of mechanical stimulation, they could potentially be entrained using mechanical means, if not chemical. Therefore, both BMSCs, used as a positive control here as these cells were previously reported to respond to mechanical stretch, and DPSCs were seeded onto fibronectin-coated, flexible silicone chambers (Figure 4(a)) and subjected to 3 days of rhythmic cyclical mechanical stretch, undergoing 12 h of cyclical stretching followed by 12 h of relaxation. After this regime was completed, samples were collected in the absence of mechanical stimulation at two opposite temporal phases at either 20 h or 32 h following their last exposure to the stretch (omitting the first 12 h to exclude any transient effects) and the clock gene expressions were analysed (Figure 4(b)), in a similar manner to the experimental design following dexamethasone exposure. Here, in striking contrast to synchronisation with dexamethasone, the clock gene levels in DPSCs appeared to peak and trough in a characteristic antiphasic manner for *Bmal1* and *Per2,* respectively; in both BMSCs and DPSCs *Bmal1* peaked in expression at 20 h, in contrast to *Per2* which peaked at the opposite circadian time point, being much higher in expression at 32 h, compared to 20 h.

**Figure 5:**
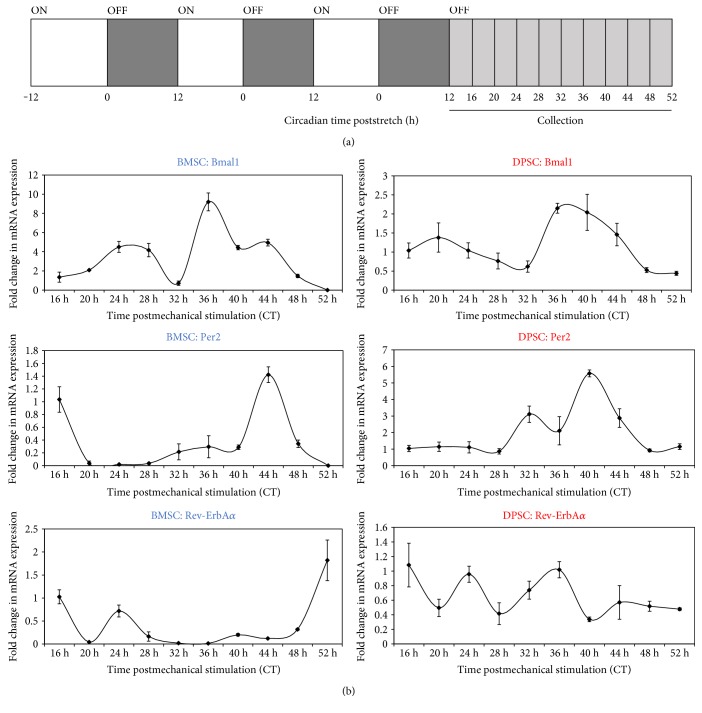
Circadian gene dynamics in human adult stem cells after rhythmic mechanical stimulation protocol. (a) Schematic diagram outlining the schedule of the 12 h ON : 12 h OFF (stretch : rest) regime followed by sample collection every 4 h between 16 h–52 h. (b) Quantitative RT-PCR analyses showing temporal expression profiles of clock genes following 3 days of rhythmic mechanical stimulation. Data are expressed as the mean +/− of ΔCt ± SEM relative to 16 h time point and normalised against the housekeeping gene *GAPDH.* Bars represent means ± SEM of 3 independent experiments.

Again, samples were then collected over a longer time course to further investigate these significant differences in gene expression and gain a finer temporal resolution over a 1.5 circadian cycle, the clock gene expressions were analysed every four hours 16 h to 52 h postrhythmic mechanical stimulation (Figure 5(a)). From these longer circadian mechanical time courses, it was evident that both DPSCs and BMSCs could produce oscillating expression of core clock genes using rhythmic mechanical stimulation. In the DPSCs, *Bmal1* expression was clearly at its trough at 32 h, which was the circadian time point at which *Per2* appeared to initially peak (Figure 5(b)). When analysed by cosinor periodogram (Table 3), *Bmal1* expression was shown to exhibit significant circadian rhythmicity in DPSCs (*p* = 0.02). Surprisingly, no circadian clock genes were found to show significant circadian rhythmicity in BMSCs.

### 3.4. The Pluripotency Marker Sox2 Shows Initial Cyclical Gene Expression following Glucocorticoid and Mechanical Synchronisation

**Figure 6:**
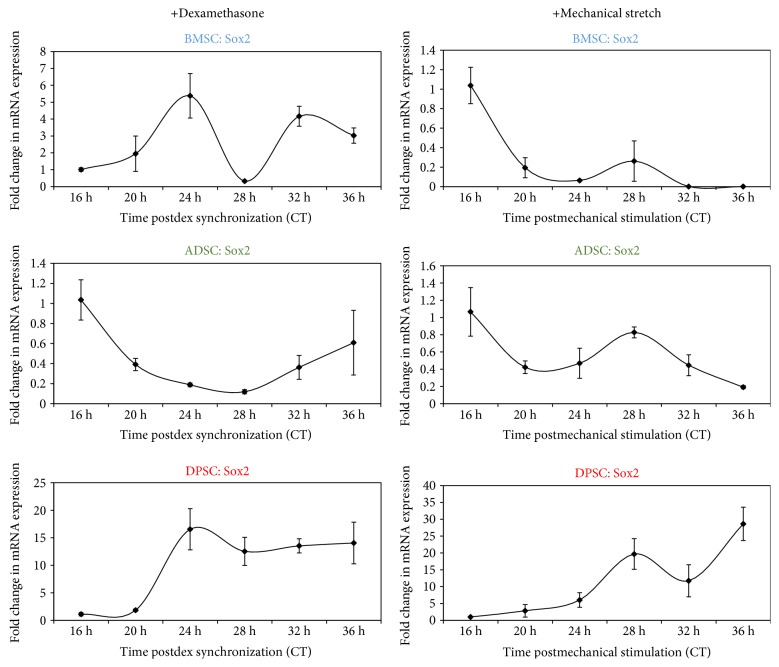
Human pluripotency marker SOX2 shows cyclical temporal gene expression in adult stem cells initially following dexamethasone or rhythmic mechanical stimulation. Quantitative RT-PCR analyses showing temporal expression profile of the pluripotency marker *Sox2* synchronisation with dexamethasone or rhythmic mechanical stimulation and collected every four hours over 1 circadian cycle (16 h–36 h). Data are expressed as the mean of ΔCt ± SEM relative to 16 h time point and normalised against the housekeeping gene *GAPDH*. Bars represent means ± SEM of 3 independent experiments.

Using both synchronisation mechanisms, it was next determined if any pluripotency stem cell markers displayed an initial circadian gene expression pattern following either glucocorticoid or rhythmic mechanical stimulation. To this end, *Sox2* was analysed and its temporal expression determined every four hours over one circadian day under the same synchronisation conditions as described above in the three stem cell types (Figure 6). *Sox2* showed initial cyclical expression in both BMSCs and ADSCs but not DPSCs following dexamethasone synchronisation. Strikingly, it was found that *Sox2* showed a very similar pattern of gene expression to that of *Rev-ErbAα* (Supplementary Fig. 2).

When analysed by cosinor periodogram (Table 4), *Sox2* expression was shown to exhibit significant initial circadian rhythmicity in ADSCs (*p* = 0.007) when exposed to dexamethasone synchronisation, whilst it was just short of significance in DPSCs following dexamethasone (*p* = 0.074) and cyclical mechanical stretch (*p* = 0.087). However, no significant circadian rhythmicity in *Sox2* could be observed in BMSCs, despite it showing a similar pattern.

## 4. Discussion

In this research, it has been shown that human stem cells derived from different human adult tissues did exhibit different levels and temporal expression patterns of core clock genes, stabilising loop genes and stem cell markers. It was also observed that the adult stem cells from different sources did indeed respond to circadian synchronising signals very differently as predicted; for instance, the BMSCs appeared to synchronise more readily in response to chemical stimulation than mechanical stimulation, but the DPSCs were much more responsive to entrainment by mechanical means. It has been experimentally evidenced that the more primitive human stem cells such as DPSCs have a different profile of the molecular circadian rhythm, in terms of both the relative levels of clock gene expression and the oscillating temporal patterns of gene expression after synchronisation by chemical means (i.e., dexamethasone exposure). It appears that the DPSCs are much less responsive to the dexamethasone synchronisation, which may be due to less developed circadian components which relay these signals. Indeed, their relative unresponsiveness to dexamethasone may be due to their early developmental origin from the migrating neural crest cells and their resulting ectomesenchymal composition. Therefore, upon DPSC differentiation, it is possible that they will gain responsiveness to circadian synchronisation and/or undergo maturation of the apparatus necessary for circadian gene oscillations. This is consistent with the research by Yagita et al. showed that embryonic stem cells do not have the capacity for circadian synchronisation by chemical means using forskolin; but upon differentiation and maturation, this ability can be gained [22]. In contrast, our results confirm previous findings that the more mature MSCs derived from the bone marrow and adipose tissue did exhibit robust clock rhythms that were responsive to glucocorticoid synchronisation and showed clear antiphasic relationships of the positive and negative arms of core TTFLs. For example, Wu et al. showed similarly oscillating expressions of clock genes in cultures of murine and human BMSCs in response to dexamethasone [19] whilst Huang et al. demonstrated that human BMSCs and ADSCs have circadian oscillations induced by serum shock [20].

As the DPSCs could not be synchronised by chemical means, a novel synchronising mechanism to which they may respond was sought. Circadian mechanosensory entrainment has been previously investigated by Simoni et al., who found that 12 h : 12 h cycles of vibration and silence, respectively, were sufficient to synchronise the daily locomotor activity of *Drosophila melanogaster* [18]. Moreover, it has recently been published that a mechanical environment of the epithelial stem cell niche within the mammalian mammary tissue controls the amplitude of the molecular clock oscillations [7]. Therefore, rhythmic mechanical stretch was utilised as an entraining factor for adult human stem cells using a uniaxial mechanical stretch apparatus. Following the stretch entrainment and subsequent analysis by cosinor periodogram, differences were observed in the phasing and period of the clock genes in the same stem cell types, highlighting how the entrainment mechanisms lead to different effects. It was observed that the DPSCs can be entrained by rhythmic mechanical stretch and appeared more responsive than the BMSCs; the *Bmal1* gene oscillations induced by rhythmic mechanical stimulation in the DPSCs resembled the temporal clock gene expression patterns seen in BMSCs in response to dexamethasone. This may be due to the fact that *in vivo*, DPSCs experience significant mechanical stimulation in the form of jaw movement, occlusion forces, and hydrostatic pressures and are one of the few stem cell niches to also experience thermal shock and extreme temperature fluctuations and so are much more likely to respond to this form of stimulation. Moreover, when the initial expression profile of the pluripotency marker *Sox2* was compared to the profile of the stabilising loop gene *Rev-ErbAα*, a strikingly similar pattern was observed following synchronisation with dexamethasone in BMSCs and ADSCs. In contrast, the rhythmic mechanical stimulation in DPSCs was able to induce the cyclical expression of the core clock genes as well as a pluripotency marker *Sox2*. These results therefore suggest a novel regulation of the *Sox2* gene which may be under both circadian and mechanical controls in different stem cell types.

The circadian clock in mammals has been extensively shown to have a key regulatory role on various tissue systems, including musculoskeletal tissues. It is therefore vital that such temporal regulation be taken into account when optimising and integrating any cellular/tissue constructs into the body, if one hopes for more successful tissue engineering strategies. For instance, it has previously been shown that the circadian regulation is significantly involved in the establishment of osseointegration under vitamin D regulation. Here, KEGG pathway analysis showed the potential association of the circadian rhythm with the success of implant osseointegration [27]. The circadian rhythm has also been shown to regulate coexisting populations of epidermal stem cells at opposite phases of the clock, which are differentially prone to activation by external stimulation [5]. Furthermore, research indicates that epidermal stem cells differ in their responsiveness to proliferation- and differentiation-like cues over a 24 h cycle [6], which may have vast implications in tissue engineering, where one cellular state may be favoured to encourage successful implant integration.

Overall, these findings suggest that the mechanism of entraining stem cell clocks by using each unique stem cell optimal synchronisation method offers an insightful way in which stem cells can be ‘primed' to respond to the desired tissue engineering applications. With this in mind, mechanical entrainment of human adult stem cells allows for a noninvasive means by which the circadian clock in human adult stem cells can be directed and controlled whilst maintaining their appropriate clock timing, thus avoiding the need for additional exogenous chemical or thermal stimuli.

## Supplementary Material

Supplementary Figure 1. Comparison of temporal clock gene expression between unsynchronised and dexamethasone-synchronised BMSCs. Quantitative RT-PCR analyses overlays of temporal clock gene expression in dexamethasone synchronised and unsynchronised (negative control) samples (NC). Data is expressed as the mean of ΔCt ± SEM calculated from the Ct value of each gene relative to the 16 h time point and normalised against the housekeeping gene *GAPDH.* Bars represent means ± SEM of 2-3 independent experiments. Supplementary Figure 2. Comparison of the cyclical expression of *Rev-ErbA* and *Sox2* following synchronisation by dexamethasone or rhythmic mechanical stretch. Quantitative RT-PCR analyses overlays of *Rev-ErbA* and *Sox2* temporal gene expressions over 1 circadian cycle (16 h to 36 h) following synchronisation with dexamethasone or mechanically. Data is expressed as the mean of ΔCt ± SEM calculated from the Ct value of each gene relative to the 16 h time point and normalised against the housekeeping gene *GAPDH.* Bars represent means ±SEM of 3 independent experiments.

## Figures and Tables

**Table 1:** qPCR primer sequences (human).

Gene	Forward (5′ to 3′)	Reverse (5′ to 3′)
*GAPDH*	CAAGGTCATCCATGACAACTTTG	GGGCCATCCACAGTCTTCTG
*Bmal1*	TGCCTCGTCGCAATTGG	ACCCTGATTTCCCCGTTCA
*Per2*	GTCCAGCCCCCACCTTTC	GGGAAGGAATAACTGGGTAGCA
*Per1*	CTCAGTGGCTGTCTCCTTCC	GAGCCAGGAGCTCAGAGAAG
*Rev-ErbAα*	CTTCAATGCCAACCATGCAT	CCTGATTTTCCCAGCGATGT
*Sox2*	GAGAGAAAGAAAGGGAGAGAAG	GAGAGAGGCAAACTGGAATC

**Table 2:** Cosinor analysis of circadian clock rhythmicity in human BMSCs, ADSCs, and DPSCs following dexamethasone synchronisation.

Cell	Gene	Period (h)	*p* value	Acrophase (°)	Amplitude	Mesor	Robustness (%)
BMSC	Bmal1^∗^	23.9	0.007	−76	0.331	0.451	76.2
	Per2	20.2	0.076	−261	1.099	1.597	52.1
	Rev-erbA	26.0	0.391	−287	1.853	3.158	23.6
ADSC	Bmal1^∗^	20.0	0.006	−289	1.264	1.885	77.4
	Per2^∗^	23.4	0.021	−98	0.502	0.486	66.6
	Rev-erbA^∗^	21.8	0.001	−314	0.611	0.914	85.1
DPSC	Bmal1	20.0	0.571	−9	0.325	0.720	14.9
	Per2	26.0	0.101	−176	1.089	1.071	47.9
	Rev-erbA	26.0	0.055	−266	4.952	4.938	56.2

^∗^Significance according to the cosinor analysis software.

**Table 3:** Cosinor analysis of circadian clock rhythmicity in human BMSCs and DPSCs following synchronisation by rhythmic mechanical stretch.

Cell	Gene	Period (h)	*p* value	Acrophase (°)	Amplitude	Mesor	Robustness (%)
BMSC	Bmal1	26.0	0.571	−346	1.754	3.568	21.6
	Per2	26.0	0.123	−51	0.428	0.343	45.0
	Rev-erbA	26.0	0.559	−171	0.316	0.397	15.4
DPSC	Bmal1^∗^	20.0	0.020	−135	0.664	1.146	67.0
	Per2	26.0	0.132	−354	1.362	2.179	43.9
	Rev-erbA	21.2	0.505	−49	0.155	0.669	17.8

^∗^Significance according to the cosinor analysis software.

**Table 4:** Cosinor analysis of pluripotency marker *Sox2* rhythmicity in human BMSCs, ADSCs, and DPSCs following synchronisation by dexamethasone and rhythmic mechanical stretch.

	Cell	Period (h)	*p* value	Acrophase (°)	Amplitude	Mesor	Robustness (%)
Dex	BMSC	26.0	0.658	−218	1.114	2.348	19.0
	ADSC^∗^	21.8	0.009	−348	0.371	0.354	74.3
	DPSC	26.0	0.074	−261	6.463	7.800	52.5
Stretch	BMSC	26.0	0.541	−61	0.328	0.279	41.2
	ADSC	26.0	0.863	−107	0.119	0.569	9.3
	DPSC	26.0	0.087	−311	9.926	11.858	55.7

^∗^Significance according to the cosinor analysis software.

## References

[1] Arthur A., Rychkov G., Shi S., Koblar S. A., Gronthos S. (2008). Adult human dental pulp stem cells differentiate toward functionally active neurons under appropriate environmental cues. *Stem Cells*.

[2] Sinanan A., Hunt N. P., Lewis M. P. (2004). Human adult craniofacial muscle-derived cells: neural-cell adhesion-molecule (NCAM; CD56)-expressing cells appear to contain multipotential stem cells. *Biotechnology and Applied Biochemistry*.

[3] Davies O. G., Cooper P. R., Shelton R. M., Smith A. J., Scheven B. A. (2015). A comparison of the in vitro mineralisation and dentinogenic potential of mesenchymal stem cells derived from adipose tissue, bone marrow and dental pulp. *Journal of Bone and Mineral Metabolism*.

[4] Stanko P., Kaiserova K., Altanerova V., Altaner C. (2014). Comparison of human mesenchymal stem cells derived from dental pulp, bone marrow, adipose tissue, and umbilical cord tissue by gene expression. *Biomedical Papers of the Medical Faculty of the University Palacky, Olomouc, Czechoslovakia*.

[5] Janich P., Pascual G., Merlos-Suárez A. (2011). The circadian molecular clock creates epidermal stem cell heterogeneity. *Nature*.

[6] Janich P., Toufighi K., Solanas G. (2013). Human epidermal stem cell function is regulated by circadian oscillations. *Cell Stem Cell*.

[7] Yang N., Williams J., Pekovic-Vaughan V. (2017). Cellular mechano-environment regulates the mammary circadian clock. *Nature Communications*.

[8] Yamazaki S., Numano R., Abe M. (2000). Resetting central and peripheral circadian oscillators in transgenic rats. *Science*.

[9] Reppert S. M., Weaver D. R. (2002). Coordination of circadian timing in mammals. *Nature*.

[10] Gekakis N., Staknis D., Nguyen H. B. (1998). Role of the CLOCK protein in the mammalian circadian mechanism. *Science*.

[11] King D. P., Zhao Y., Sangoram A. M. (1997). Positional cloning of the mouse circadian clock gene. *Cell*.

[12] Kume K., Zylka M. J., Sriram S. (1999). mCRY1 and mCRY2 are essential components of the negative limb of the circadian clock feedback loop. *Cell*.

[13] Shearman L. P., Sriram S., Weaver D. R. (2000). Interacting molecular loops in the mammalian circadian clock. *Science*.

[14] Guillaumond F., Dardente H., Giguère V., Cermakian N. (2005). Differential control of Bmal1 circadian transcription by REV-ERB and ROR nuclear receptors. *Journal of Biological Rhythms*.

[15] Guo B., Chatterjee S., Li L. (2012). The clock gene, brain and muscle Arnt-like 1, regulates adipogenesis *via* Wnt signalling pathway. *The FASEB Journal*.

[16] Shimba S., Ishii N., Ohta Y. (2005). Brain and muscle Arnt-like protein-1 (BMAL1), a component of the molecular clock, regulates adipogenesis. *Proceedings of the National Academy of Sciences of the United States of America*.

[17] Silver R., LeSauter J., Tresco P. A., Lehman M. N. (1996). A diffusible coupling signal from the transplanted suprachiasmatic nucleus controlling circadian locomotor rhythms. *Nature*.

[18] Simoni A., Wolfgang W., Topping M. P., Kavlie R. G., Stanewsky R., Albert J. T. (2014). A mechanosensory pathway to the Drosophila circadian clock. *Science*.

[19] Tirkkonen L., Halonen H., Hyttinen J. (2011). The effects of vibration loading on adipose stem cell number, viability and differentiation towards bone-forming cells. *Journal of the Royal Society Interface*.

[20] Wu X., Yu G., Parks H. (2008). Circadian mechanisms in murine and human bone marrow mesenchymal stem cells following dexamethasone exposure. *Bone*.

[21] Huang T. S., Grodeland G., Sleire L., Wang M. Y., Kvalheim G., Laerum O. D. (2009). Induction of circadian rhythm in cultured human mesenchymal stem cells by serum shock and cAMP analogs in vitro. *Chronobiology International*.

[22] Yagita K., Horie K., Koinuma S. (2010). Development of the circadian oscillator during differentiation of mouse embryonic stem cells in vitro. *Proceedings of the National Academy of Sciences*.

[23] Paulose J. K., Rucker E. B., Cassone V. M. (2015). Analysis of circadian rhythms in embryonic stem cells. *Stem Cell Protocols*.

[24] Balsalobre A., Brown S. A., Marcacci L. (2000). Resetting of circadian time in peripheral tissues by glucocorticoid signaling. *Science*.

[25] Sun L., Qu L., Zhu R. (2016). Effects of mechanical stretch on cell proliferation and matrix formation of mesenchymal stem cell and anterior cruciate ligament fibroblast. *Stem Cells International*.

[26] O’Cearbhaill E. D., Punchard M. A., Murphy M., Barry F. P., McHugh P. E., Barron V. (2008). Response of mesenchymal stem cells to the biomechanical environment of the endothelium on a flexible tubular silicone substrate. *Biomaterials*.

[27] Mengatto C. M., Mussano F., Honda Y., Colwell C. S., Nishimura I. (2011). Circadian rhythm and cartilage extracellular matrix genes in osseointegration: a genome-wide screening of implant failure by vitamin D deficiency. *PLoS One*.

