# Supplementary material for: Comparing Circadian Dynamics in Primary Derived Stem Cells from Different Sources of Human Adult Tissue
Source: Stem Cells Int. 2017 Oct 23;2017:2057168. doi: 10.1155/2017/2057168 (PMC5672638; doi:10.1155/2017/2057168)
Supplement: Supplementary file 1 — Supplementary Figure 1. Comparison of temporal clock gene expression between unsynchronised and dexamethasone-synchronised BMSCs. Quantitative RT-PCR analyses overlays of temporal clock gene expression in dexamethasone synchronised and unsynchronised (negative control) samples (NC). Data is expressed as the mean of ΔCt ± SEM calculated from the Ct value of each gene relative to the 16 h time point and normalised against the housekeeping gene GAPDH. Bars represent means ± SEM of 2-3 independent experiments. Supplementary Figure 2. Comparison of the cyclical expression of Rev-ErbA and Sox2 following synchronisation by dexamethasone or rhythmic mechanical stretch. Quantitative RT-PCR analyses overlays of Rev-ErbA and Sox2 temporal gene expressions over 1 circadian cycle (16 h to 36 h) following synchronisation with dexamethasone or mechanically. Data is expressed as the mean of ΔCt ± SEM calculated from the Ct value of each gene relative to the 16 h time point and normalised against the housekeeping gene GAPDH. Bars represent means ±SEM of 3 independent experiments. [file 2057168.f1.pdf]

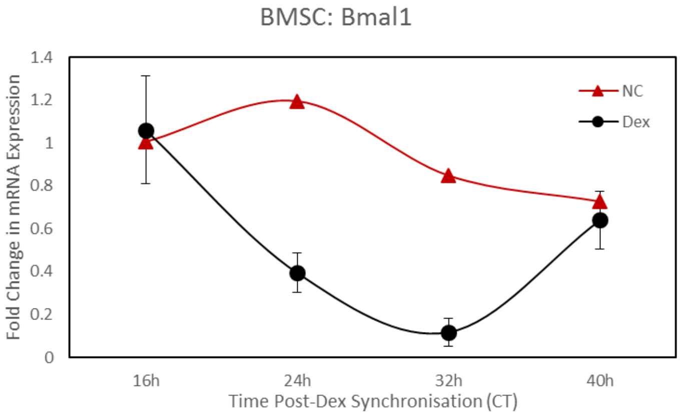

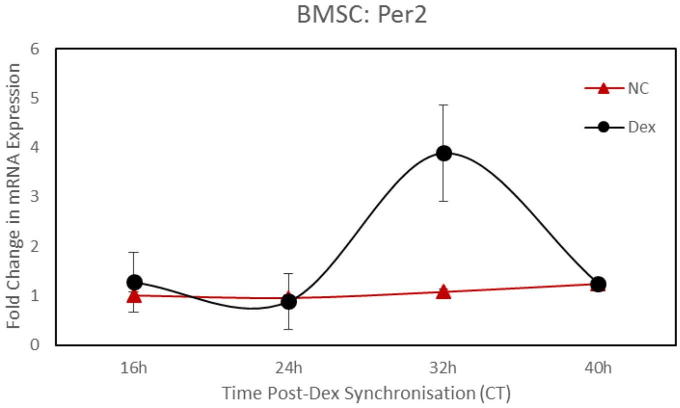

Supplementary Fig 1. Comparison of temporal clock gene expression between unsynchronised and dexamethasone-synchronised BMSCs. Quantitative RT-PCR analyses overlays of temporal clock gene expression in dexamethasone synchronised and unsynchronised (negative control) samples (NC). Data is expressed as the mean of ΔCt ±SEM calculated from the Ct value of each gene relative to the 16 h time point and normalised against the housekeeping gene *GAPDH.* Bars represent means ±SEM of 2-3 independent experiments.

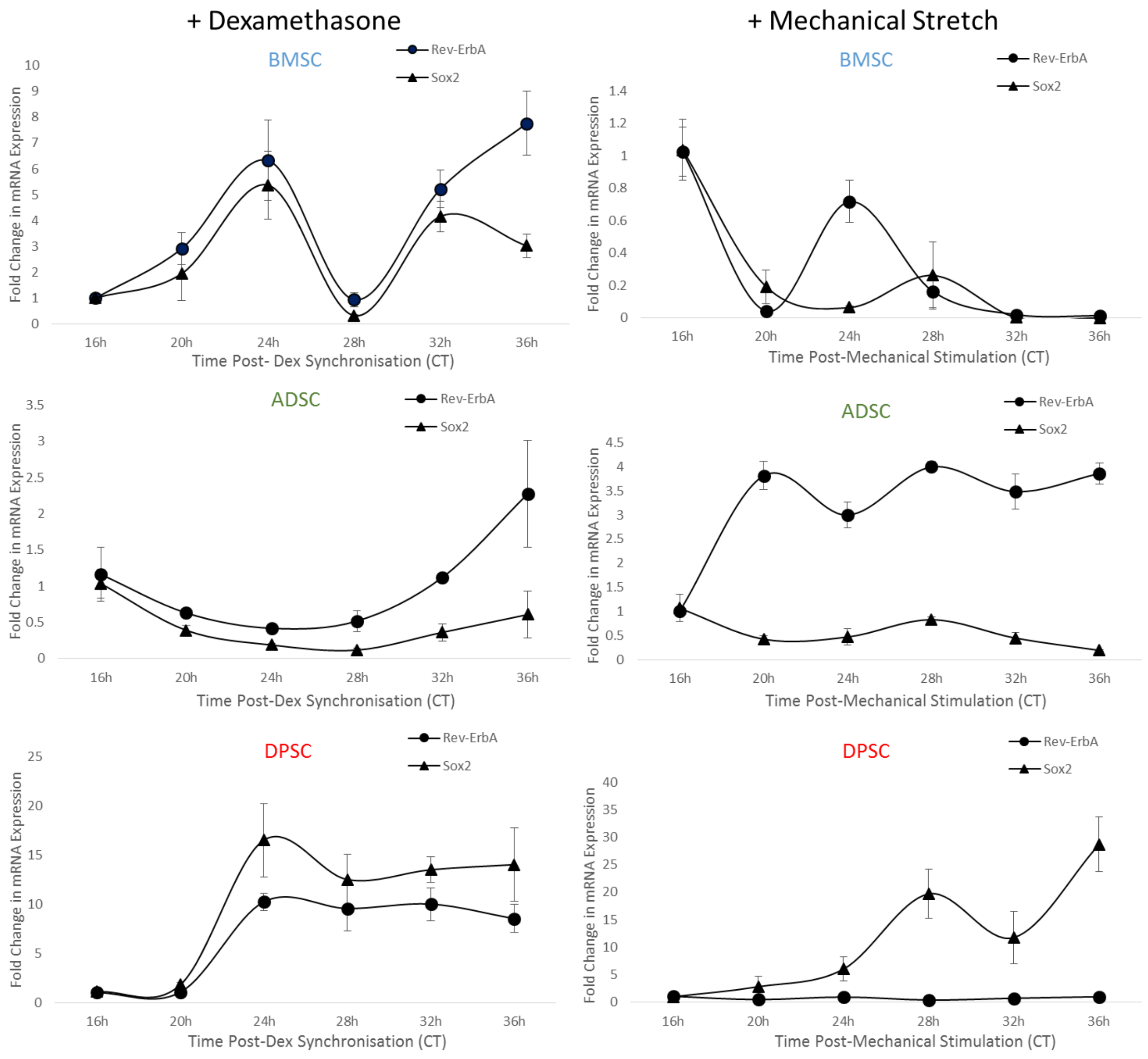

Supplementary Fig 2. Comparison of the cyclical expression of *Rev-ErbA* and *Sox2* following synchronisation by dexamethasone or rhythmic mechanical stretch. Quantitative RT-PCR analyses overlays of *Rev-ErbA* and *Sox2* temporal gene expressions over 1 circadian cycle (16 h to 36 h) following synchronisation with dexamethasone or mechanically. Data is expressed as the mean of ΔCt ±SEM calculated from the Ct value of each gene relative to the 16 h time point and normalised against the housekeeping gene *GAPDH.* Bars represent means ±SEM of 3 independent experiments.
